# Factors Associated with Postoperative Prolonged Mechanical Ventilation in Pediatric Liver Transplant Recipients

**DOI:** 10.1155/2017/3728289

**Published:** 2017-07-03

**Authors:** Olubukola O. Nafiu, Katari Carello, Anjana Lal, John Magee, Paul Picton

**Affiliations:** ^1^Department of Anesthesiology, University of Michigan, Ann Arbor, MI, USA; ^2^Section of Pediatric Anesthesiology, University of Michigan, Ann Arbor, MI, USA; ^3^Department of Surgery, University of Michigan, Ann Arbor, MI, USA

## Abstract

**Introduction:**

Almost all pediatric orthotopic liver transplant (OLT) recipients require mechanical ventilation in the early postoperative period. Prolonged postoperative mechanical ventilation (PPMV) may be a marker of severe disease and may be associated with morbidity and mortality. We determined the incidence and risk factors for PPMV in children who underwent OLT.

**Methods:**

This was a retrospective analysis of data collected on 128 pediatric OLT recipients. PPMV was defined as postoperative ventilation ≥ 4 days. Perioperative characteristics were compared between cases and control groups. Multivariable logistic regression analysis was used to calculate odds ratios for PPMV after controlling for relevant cofactors.

**Results:**

An estimated 25% (95% CI, 17.4%–32.6%) required PPMV. The overall incidence of PPMV varied significantly by age group with the highest incidence among infants. PPMV was associated with higher postoperative mortality (*p* = 0.004) and longer intensive care unit (*p* < 0.001) and hospital length of stay (*p* < 0.001). Multivariable analysis identified young patient age, preoperative hypocalcemia, and increasing duration of surgery as independent predictors of PPMV following OLT.

**Conclusion:**

The incidence of PPMV is high and it was associated with prolonged ICU and hospital LOS and higher posttransplant mortality. Surgery duration appears to be the only modifiable predictor of PPMV.

## 1. Introduction

The vast majority of children undergoing orthotopic liver transplantation (OLT) are maintained on mechanical ventilation (MV) in the immediate postoperative period [1, 2]. Reasons for this practice include concerns about graft function, postoperative respiratory depression from opioids, preexisting malnutrition, and organ-recipient size mismatch as well as poor cooperation of young children with postoperative instructions [3]. With current improvements in the perioperative care of liver transplant recipients, there is increasing drive towards early extubation in both adults [4, 5] and children [3, 6].

Although many children require only a few days of MV following OLT, for some a more prolonged course is necessary. In general, prolonged postoperative mechanical ventilation (PPMV) in intensive care unit (ICU) patients is a marker of severe adverse events and is associated with higher morbidity and mortality as well as extraordinary resource utilization [3, 7]. Patients requiring PPMV have survived the acute phase of surgery but spend an increased amount of time in the ICU, consume about 50% of all intensive care unit (ICU) resources, and are more likely to die [7]. Consequently, investigating the incidence and factors predisposing to PPMV following liver transplant is an important area of research with the potential to reduce cost of care and improve long-term outcome for these patients.

To our knowledge, factors associated with PPMV following pediatric liver transplantation have not been comprehensively characterized. Here, we investigate the incidence and risk factors for PPMV in children who underwent liver transplant at a US tertiary hospital. We also explored the relationship between PPMV and a number of clinically important outcomes such as duration of intensive care unit (ICU) and total hospital length of stay (LOS) as well as 1-year posttransplant mortality rates.

## 2. Methods

This was a retrospective cohort study. Following Institutional Review Board approval* (IRB number HUM00068847), *we queried our perioperative, clinical information system (Centricity® General Electric Healthcare, Waukesha, Wisconsin) and the Organ Transplantation Information System (OTIS) database. The records of all children with age ≤ 17 yr who underwent OLT at our institution were examined. We extracted the following clinical and anthropometric information from the database: age, gender, weight, American Society of Anesthesiology (ASA) physical status, and etiology of liver failure. All preoperative laboratory results were obtained from the blood drawn for preoperative evaluation on the day of operation.

### 2.1. Case Definition and Variable Transformations

Definition of PPMV following pediatric liver transplant is unavailable. Adult publications often utilize a 48 hr cut-point [8, 9]. This may not necessarily be a practical cut-point in children. Therefore, to estimate the incidence of PPMV, we used previously described cut-point which indicates that ≥96 hr mechanical ventilation was a marker of disease severity in pediatric intensive care unit (PICU) patients [10] to transform days of postoperative mechanical ventilation to a categorical variable (PPMV yes/no). Thus, the primary outcome (dependent) variable for this report, PPMV, was defined as requirement of mechanical ventilation for ≥4 consecutive postoperative days. This value also matched the 75th percentile number of days of postoperative ventilation in our study cohort.

Anesthesia duration was measured as the interval between documented “patient in room” and “anesthesia end.” Similarly, duration of surgery was measured from the beginning of “surgical incision” to “surgery end.” Surgery and anesthesia durations were considered prolonged if the times were ≥453 min and 592 min, respectively (each value corresponding to the 75th percentile times for the study cohort). Finally, length of stay (LOS) was calculated from the day of transplantation to ICU or hospital discharge. Mortality data were assessed as death occurring within one year from the day of liver transplantation.

### 2.2. Exclusion Criteria

Patients who required mechanical ventilation during the 48 hr preceding surgery (ventilator-dependent) were excluded. We also excluded patients who were older than 17 yr at the time of transplantation. Similarly, patients who died either in the operating room or within 96 hr of surgery were excluded. Including these patients in the analysis would result in a misclassification bias because they would have been included in the non-PMV group. Present analysis also excluded patients who were reintubated within 48 hr of successful extubation because reintubation is a known risk factor for prolonged intubation [10].

### 2.3. Statistical Analysis

Data analyses were carried out with Statistical Package for the Social Sciences (SPSS) for Windows, version 22.0, (SPSS Inc., Chicago, IL). Basic descriptive statistics including means, standard deviations, and percentages were calculated for demographic and anthropometric data. Distribution of skewed continuous variables such as age and hospital and ICU LOS was displayed with box and whisker plots after stratifying patients according to postoperative ventilation status. When necessary, continuous variables were transformed to categorical variables to emphasize clinical applicability. Pearson's Chi-square test for categorical variables and Student's *t*-test and one-way ANOVA for continuous variables were used to examine baseline clinical and perioperative factors associated with PMV. Prevalence of preoperative anemia, malnutrition, hyponatremia, and hypoalbuminemia were described as simple proportions. Furthermore, intraoperative uses of blood products (packed red blood cells, fresh frozen plasma, platelets, and cryoprecipitate) were described as simple proportions and compared across postoperative ventilation groups. We also explored the association between preoperative anemia and use of intraoperative blood transfusion.

#### 2.3.1. Multivariable Logistic Regression Analysis

Multivariable analysis was performed using logistic regression (backward stepwise method) to calculate the adjusted odds ratios for the occurrence of PPMV.

To fit the logistic regression model, we estimated Pr(*Y* = 1∣*X*), where *Y* is the binary dependent variable and *X* is the vector of the covariates. Derivation of the response variable *Y* (PPMV yes/no) is as detailed above. Model covariates were selected based on statistical significance or clinical relevance. Predictor variables were treated as continuous or categorical as needed. For example, to align with clinical utility, preoperative serum calcium and albumin were transformed into categorical variables (hypocalcemia or hypoalbuminemia, resp.). Similarly, intraoperative blood product utilization was coded as yes/no categorical variables for the regression model. Prior to performing multiple logistic regression analyses, variables were examined for multicollinearity by first creating a correlation matrix and scanning for highly correlated variables (≥0.7). After collinearity diagnostics, all variables were entered into a nonparsimonious logistic regression model to determine independent predictors of PMV. All variables with a *p* value ≤ 0.05 were deemed independent predictors of PMV. Goodness of fit of the model was assessed using the Hosmer-Lemeshow test [11]. Furthermore, the model's predictive value was assessed using receiver operating characteristic (ROC) analyses and area under the curve (AUC). All reported *p* values were 2-sided and a *p* value of <0.05 was considered as statistically significant.

## 3. Results

**Figure 1:**
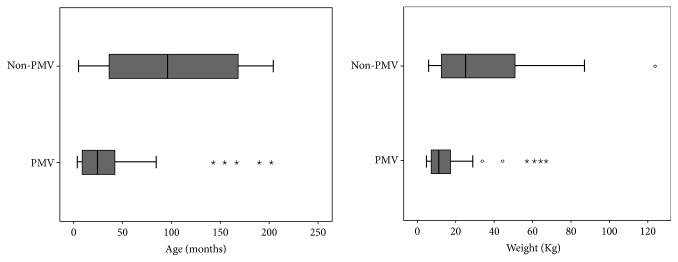
Box plots showing the distribution of age (months) and weight (Kg) stratified by postoperative ventilation status among 128 pediatric liver transplant recipients. Expectedly age and weight data were skewed. Children requiring prolonged postoperative ventilation were significantly younger (*p* = 0.001) and weighed less (*p* = 0.005) than those who were extubated early. PMV: prolonged mechanical ventilation. Circles and stars refer to outliers or extreme values.

One hundred and twenty-eight patients aged 4 mth to 17 yr who underwent liver transplant were included in this analysis. There were 22 (17.2%) infants, 37 (28.9%) preschool children, and 69 (53.9%) older children. The median duration of postoperative ventilation days for the total sample was 2 (1,5) days. Overall, 32 (25%) patients required PPMV. Age and weight distribution in the study cohort displayed a slight right skew (Figure 1) with children who required PPMV being significantly younger (*p* < 0.001) and weighing less (*p* = 0.005) than those in the non-PPMV group (Figure 1).

Table 1 summarizes the baseline preoperative clinical and laboratory variables between the groups. In bivariable analyses, we identified a number of differences in the two study groups. Patients in the PPMV group were more likely to be infants and weigh below 10 kg. They were also more likely to have a preoperative liver diagnosis of biliary atresia. Furthermore, mean preoperative sodium, total protein, albumin, and calcium were significantly lower among children who required PMV. Similarly, preoperative WBC count, liver enzymes, and PT values were significantly higher in the PMV than in the non-PPMV group. Although baseline anemia was common in the study cohort, it was not significantly associated with PPMV.

Intraoperative and postoperative variables stratified by group allocation are detailed in Table 2. Mean duration of anesthesia and surgery was significantly longer in the PPMV compared to the non-PPMV group (Table 2). Intraoperative blood product utilization was very common in this group of patients, although only the use of fresh frozen plasma (FFP) and cryoprecipitate was significantly associated with PPMV (Table 2). Expectedly, intraoperative PRBC transfusion rates were significantly higher in patients with preoperative anemia compared to the nonanemic group (95.1% versus 78.4%; *p* = 0.022). Transfusion rates for FFP, platelets, and cryoprecipitate were similarly higher in the anemic group.

**Figure 2:**
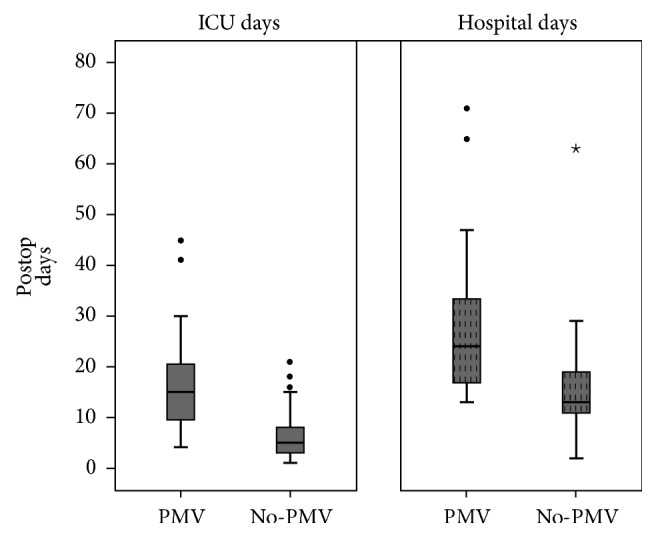
Box plot showing the distribution of postoperative ICU and hospital length of stay stratified according to duration of postoperative ventilation status. Patients in the PMV group had significantly longer ICU and hospital LOS compared with the non-PMV group. ICU: intensive care unit; LOS: length of stay; PMV: prolonged mechanical ventilation. Circles and stars refer to outliers or extreme values.

Total intensive care unit (ICU) and hospital LOS displayed a leptokurtic right skew (Figure 2). Mean ICU LOS was ten days longer in the PPMV group compared with that in those who were extubated early (16.4 days versus 6.6 days; *p* < 0.001). Similarly, mean posttransplant hospital LOS was 12 days longer among children requiring PPMV compared to those who did not (27.7 days versus 15.2 days; *p* < 0.001). Overall posttransplant 1-year mortality was 5.5%. Children in the PPMV group had 8 times higher unadjusted odds of mortality compared with the control group (15.6% versus 2.1%, OR = 8.5; 95% CI = 11.6–47.3; *p* = 0.011).

### 3.1. Multivariable Analyses

**Figure 3:**
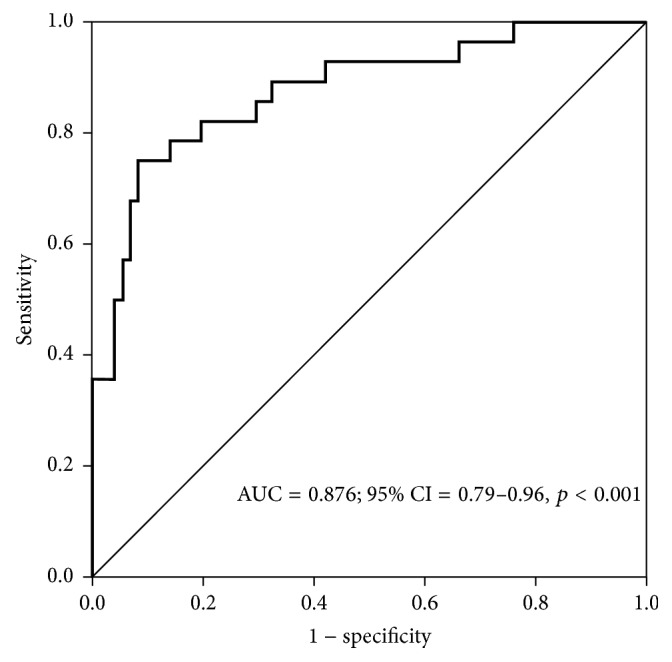
Receiver operator characteristic (ROC) curve evaluating the sensitivity and specificity of the model predicting PPMV. Area under the curve (AUC) for the predictors was 0.87 (95%  CI = 0.79–0.96; *p* < 0.001). AUC for the ROC indicates the usefulness of a test (our model) in predicting a binomial outcome (PMV yes/no). A value of 0.87 indicates “good” model predictive ability.

In order to determine predictors of PMV, we performed multiple logistic regression analysis (backward stepwise method) with PMV as the dichotomous dependent variable and many of the baseline clinical and laboratory factors found to be significantly different in Tables 1 and 2 as independent (predictor) variables. Because duration of surgery and duration of anesthesia were highly correlated (*r* = 0.91; *p* < 0.001), only the former was included in the regression model. Furthermore, the number of variables included in our regression model was hampered by the overall sample size. Therefore, despite using the backward selection method, we had to utilize clinical judgement to select the most parsimonious model. Model performance was assessed with the Hosmer-Lemeshow (HL) goodness-of-fit test as well as receiver operating characteristic curve (ROC) analysis (reported as *c*-statistic) and the HL test revealed a good model fit to our data (*χ*^2^ = 10.22; df = 8; *p* = 0.35). The *c*-statistic for the model was 0.82 (95% CI, 0.72–0.92), indicating good model discriminant ability. Furthermore, ROC curve analysis of the model's discriminant ability was good (Figure 3). The results of the multivariable logistic regression analysis are detailed in Table 3 and include the odds ratios for the initial and final models after backward exclusion. In the final model, under-five age group, increasing duration of surgery (per hour), high ASA status, and preoperative hypocalcemia were the most consistent factors associated with PMV. Specifically, for every additional hour of operation, the odds of PMV increased by 1.88. Similarly, under-five age group was associated with 9.5 times increased odds of PMV in this cohort of patients. Preoperative hypocalcemia was also a significant predictor of PMV in our patients.

## 4. Discussion

We presented comprehensive data on the incidence and risk factors as well as clinically important outcomes associated with PMV following pediatric OLT. We have demonstrated that the incidence of PMV (≥4 days of postoperative mechanical ventilation) was high in this sample of children who underwent OLT. We also identified preoperative factors including young recipient age, prolonged duration of surgery, and preoperative hypocalcemia as being significantly associated with PMV. We further demonstrated that PMV was associated with considerably increased ICU and total hospital LOS and it is significantly associated with increased posttransplant mortality.

Pediatric liver transplantation has become an effective and widely accepted treatment for severe liver disease [12]. With improvement in anesthetic and surgical management in the last 25 yr, many pediatric OLT recipients are able to resume and maintain spontaneous ventilation soon after surgery and typically only require a short period of postoperative mechanical ventilation. In children requiring PPMV, this may be due to the occurrence of a severe perioperative complication, which may be organ-specific or systemic. One of the primary objectives of the present investigation was to document the incidence of PPMV in pediatric OLT recipients and we found that, despite using a liberal definition of PPMV (≥4 days), the incidence was about 25%. Although this percentage appears to be quite high (and may reflect local practices), other investigators [13] have reported rates as high as 31.6% among adult OLT recipients who developed postoperative pneumonia. There is presently no comparable published data describing PPMV among pediatric OLT recipients. The present report therefore addresses key research needs on the subject of pediatric solid organ transplantation and it represents the necessary first steps towards delineating the scope of the problem. Large multicenter studies are necessary in order to compare practice across transplant units and determine median duration of postoperative mechanical ventilation in OLT recipients. This should help guide clinical practice and development of future pediatric OLT standards.

A second objective of the present analysis was to identify perioperative factors that are significantly associated with PPMV in pediatric OLT recipients. To this end, we found that patient's age was the strongest predictor of PPMV after adjusting for several clinical covariates. Although the confidence interval associated with the odds ratio for age group as a risk factor for PMV was very wide, under-five children had about 10 times increased odds of PPMV compared to their older peers in our regression model. This is an important point to consider when planning OLT in under-five children. They are a group that could be targeted for specific interventions to reduce the likelihood of PPMV.

In order to effectively prevent PPMV in children undergoing OLT, it is important to recognize factors that may predispose patients to this complication. Unfortunately, similar to the findings of previous investigators reporting on other types of surgical procedures [14, 15], many of the factors identified in our study are fairly intuitive but not necessarily modifiable. For example, factors such as under-five age group, preoperative hypocalcemia, prolonged duration of anesthesia, and surgery and intraoperative blood product transfusion are either part of the pathological process or indicative of severity of the underlying disease. However, despite these limitations, presence of these risk factors in a pediatric liver transplant patient may prove useful for alerting clinicians about the need for close follow-up and early intervention. Furthermore, identification of nonmodifiable factors may be helpful for risk stratification of patients and appropriate allocation of resources.

Most published data indicate that PPMV is associated with increased postoperative morbidity and mortality as well as significant medical expenditure. We found, as did previous investigators [16, 17], that one-year posttransplant death rate among patients requiring PPMV was higher than that in those who are not. It must be noted though that it is impossible to determine a causal relationship between PPMV and mortality from retrospective case-control data such as that we used in the present report. We also provided data for the first time in a pediatric OLT cohort, spotlighting the impact of PPMV on postoperative ICU and hospital LOS following liver transplant. Our results indicate that PPMV increased mean ICU and hospital LOS by 10 and 14 days, respectively. Although we do not have data on the charges associated with posttransplant complication in the present report, the total hospital cost associated with PPMV in the pediatric liver transplant is likely very high. Increased awareness among clinicians about the factors associated with PPMV among pediatric liver transplant patients is likely to lead to targeted interventions to reduce this complication.

Although it is impossible to determine the mechanisms underlying PPMV from a retrospective study such as ours, several putative mechanisms have been highlighted by previous reviews of the subject [18, 19]. Among these are increased respiratory demands due to pulmonary edema and pleura effusion and increased intrathoracic pressure from graft-size mismatch. Other possible causes include excessive sedation, preoperative malnutrition, and electrolyte abnormalities.

We also found that children undergoing primary liver transplant who have lower or deranged values of common metabolic variables were significantly more likely to require PPMV than those with normal values of these metabolic variables. This may simply reflect a more severe degree of preoperative hepatic dysfunction in children with lower values of these variables. Lower serum levels of biochemical parameters such as albumin and sodium may be associated with expanded total body water which may in turn lead to increased lung water [3]. This could make weaning from mechanical ventilation difficult in the postoperative period. These findings agree with those of previous investigators [20] demonstrating that electrolyte abnormalities such as hypocalcemia, hypomagnesaemia, and hypokalemia may be associated with respiratory muscle dysfunction, which could in turn predispose to longer requirement of ventilator support. Further studies to determine the mechanisms underlying these observations are warranted.

## 5. Study Limitations

This study has several strengths including single institution data, which ensures consistency of practice and robustness of the data collection process and the unambiguous, categorical outcome variable. However, there are some limitations to the study. The retrospective, cross-sectional nature of the study design did not dictate standardized care during the study period. Therefore, we cannot ascribe causality to any of the factors such as mortality and length of stay associated with PPMV in the present study. Also, we do not have data on several factors (including graft weight to recipient weight, history of obstructive sleep apnea, postoperative sedation status, postoperative pulmonary complications, and postoperative analgesia method) which could affect postoperative ventilation. However, whatever the final common pathway leading to PMV is, it is an important complication that is attended by significant morbidity and mortality in the pediatric OLT patient. To this end, PPMV may be a useful outcome measure for prognostication and quality improvement initiatives in pediatric liver transplant recipients.

In conclusion, we present the first pediatric data among recipients of OLT, which indicates a high incidence of PPMV among these patients. We also identified factors such as under-five age group, prolonged duration of surgery, and preoperative hypocalcemia as independent predictors of PPMV. Additionally, this article may be the first to describe increased postoperative ICU and hospital LOS following pediatric OLT. This important association has the potential for a multiplicative effect on the total cost associated with pediatric liver transplantation. Specific strategies to reduce the incidence of PPMV in the pediatric OLT patients are highly desirable. Furthermore, mechanisms underlying this important complication deserve further elucidation.

## Figures and Tables

**Table 1:** Baseline clinical and laboratory characteristics of the study cohort by PPMV status.

Variables	All	No	Yes	*p* value
	(*N* = 128)	(*N* = 96)	(*N* = 32)	
Male sex (%)	53.9	51.0	62.1	0.260
Infants (%)	17.2	11.4	37.5	0.001
Under five (%)	51.1	37.7	77.4	<0.001
Weight (Kg)	28.5 ± 24.4	33.9 ± 25.6	18.6 ± 18.4	0.003
Height (cm)	110.6 ± 38.6	119.2 ± 36.8	91.5 ± 36.0	0.002
BMI	19.9 ± 5.3	20.2 ± 5.6	19.5 ± 4.5	0.597
Under 10 kg (%)	26.0	16.7	50.0	0.001
FTT (%)	16.0	11.1	28.6	0.032
Preoperative anemia (%)	43.0	40.3	50.8	0.500
Hepatopulmonary synd.	8.5	4.9	15.2	0.09
ASA > III (%)	61	51.4	85.7	0.001
PELD/MELD score	20.10 ± 9.49	20.13 ± 10.1	20.04 ± 7.8	0.996
*Liver disease etiology (%)*				0.014
Biliary atresia	33.0	23.0	51.5	
Neonatal cholestasis	13.8	14.8	12.1	
Hepatitis	17.0	16.4	18.2	
Metabolic diseases	12.8	12.8	0.0	
Others^*∗*^	23.4	26.2	18.2	
*Preoperative labs*				
Sodium_(mEq/L)_	137.4 ± 4.0	138.2 ± 3.8	135.7 ± 3.7	0.004
Potassium_(mEq/L)_	3.9 ± 0.5	3.9 ± 0.5	4.1 ± 0.48	0.315
Serum CO_2__(mEq/L)_	23.2 ± 3.3	24.0 ± 3.1	21.7 ± 3.1	<0.001
Calcium	8.9 ± 0.85	9.1 ± 0.9	8.6 ± 0.6	0.007
Magnesium	2.0 ± 0.3	2.0 ± 0.3	1.9 ± 0.3	0.411
Albumin_(g/dL)_	3.3 ± 0.7	3.5 ± 0.7	3.0 ± 0.5	0.007
Protein_(g/dL)_	6.4 ± 1.1	6.6 ± 0.9	5.8 ± 1.2	0.004
ALT_(IU/L)_	188.5 ± 323.3	177.4 ± 314.5	209.9 ± 343.8	0.652
AST_(IU/L)_	308.3 ± 574.0	228.7 ± 337.3	510.0 ± 929.1	0.029
Alk phos.	592.4 ± 496.8	522.5 ± 436.4	769.7 ± 597.2	0.025
PT	16.3 ± 8.6	15.5 ± 7.9	18.5 ± 9.9	0.047
PTT	38.7 ± 16.5	36.6 ± 14.0	44.0 ± 21.2	0.047
BUN	11.8 ± 5.9	11.8 ± 5.8	11.4 ± 6.3	0.706
Creatinine_(mg/dL)_	0.44 ± 0.2	0.49 ± 0.3	0.30 ± 0.2	0.001
WBC	7.8 ± 4.1	7.1 ± 3.4	9.5 ± 5.2	0.009

PPMV: prolonged postoperative mechanical ventilation; BMI: body mass index; FTT: failure to thrive; ASA: American Society of Anesthesiologists; MELD: Model for End-Stage Liver Disease; Alk Phos.: alkaline phosphate; BUN: blood urea nitrogen; WBC: white blood cells count. ^*∗*^Others: refers to other types of liver disease.

**Table 2:** Perioperative variables stratified according to PPMV status.

Variables	All patients	No	No	*p* value
	(*N* = 128)	(*N* = 96)	(*N* = 32)	
Operation time (min)	397.0 ± 83.9	377.4 ± 86.1	435.6 ± 64.9	<0.001
Anesthesia time (min)	538.1 ± 97.4	517.5 ± 99.4	578.7 ± 80.3	0.004
PRBC (cc/kg)	52.65	41.08	82.38	0.039
FFP (cc/kg)	53.57	38.41	92.55	<0.001
Platelets (cc/kg)	5.96	4.61	9.47	0.092
Crystalloid (cc/kg)	69.12	58.78	95.71	0.157
OR inotropes used	8.5	8.2	9.1	0.882
Split liver used (%)	31.0	23.6	50.0	0.016
ICU LOS (days)	9.9 ± 8.0	6.6 ± 4.4	16.6 ± 9.5	<0.001
Hospital LOS (days)	19.5 ± 12.4	15.3 ± 8.7	27.7 ± 14.2	<0.001
Reexploration	37.0	25.0	67.9	<0.001

PPMV: prolonged postoperative mechanical ventilation; PRBC: packed red blood cells; FFP: fresh frozen plasma; ICU LOS: intensive care unit length of stay; OR: operating room.

**Table 3:** Multivariable logistic regression model and parameters for factors associated with PPMV (*N* = 128).

Predictors	*β*	SE	OR	95% CI	*p* value
*Beginning model*					
Constant	−5.5	1.8	0.004	—	0.005
Under 5 yr	2.96	1.07	19.44	2.37–158.97	0.003
ASA ≥ 3	1.45	0.67	4.67	1.14–15.82	0.03
Weight (Kg)	−0.032	0.02	1.03	0.97–1.09	0.24
Sodium	−0.03	0.07	0.97	0.28–2.61	0.73
WBC	0.12	0.07	1.12	0.96–1.30	0.14
Hypocalcemia (yes/no)	1.42	0.72	4.14	1.22–15.51	0.024
Surgery duration (hr.)	0.71	0.23	2.03	1.27–3.26	0.003
FFP transfusion (yes/no)	0.88	0.98	2.43	0.35–16.68	0.36
Cryoprecipitate (yes/no)	0.50	0.71	1.65	0.41–6.68	0.48
*Final model*					
Constant	−6.8	1.66	0.001	—	<0.001
Hypocalcemia (yes/no)	1.46	0.62	4.32	1.27–13.90	0.019
Surgery duration (hr.)	0.63	0.20	1.88	1.25–2.81	0.013
ASA status ≥ 3	1.24	0.65	4.42	1.29–16.69	0.019
Under 5 yr	2.29	0.62	9.51	2.75–32.55	<0.001

PPMV: prolonged postoperative mechanical ventilation; FFP: fresh frozen plasma; ASA: American Society of Anesthesiologists; OR: odds ratio; SE: standard error.
